# Peer supervision experiences of drug sellers in a rural district in East-Central Uganda: a qualitative study

**DOI:** 10.1186/s12936-020-03343-0

**Published:** 2020-07-25

**Authors:** Arthur Bagonza, Henry Wamani, Stefan Peterson, Andreas Mårtensson, Milton Mutto, David Musoke, Freddy Eric Kitutu, David Mukanga, Linda Gibson, Phyllis Awor

**Affiliations:** 1 Department of Community Health and Behavioral Sciences, Makerere University College of Health Sciences, School of Public Health, Kampala, Uganda; 2 Department of Health Policy Planning and Management, Makerere University College of Health Sciences, School of Public Health, Kampala, Uganda; 3 International Maternal and Child Health Unit, Department of Women’s and Children’s Health, Uppsala University, Uppsala, Sweden; 4 Department of Disease Control and Environmental Health, Makerere University College of Health Sciences, School of Public Health, Kampala, Uganda; 5 Department of Pharmacy, School of Health Sciences, Makerere University College of Health Sciences, Kampala, Uganda; 6 Bill and Melinda Gates Foundation, Washington, USA; 7 School of Social Sciences, Nottingham Trent University, Nottingham, UK

**Keywords:** Drug shops, Drug sellers, Pneumonia, Malaria, Diarrhoea, Peer supervision, Uganda

## Abstract

**Background:**

Support supervision improves performance outcomes among health workers. However, the national professional guidelines for new licenses and renewal for Class C drug shops in Uganda prescribe self-supervision of licensed private drug sellers. Without support supervision, inappropriate treatment of malaria, pneumonia and diarrhoea among children under 5 years of age continues unabated. This study assessed experiences of drug sellers and peer supervisors at the end of a peer supervision intervention in Luuka District in East Central Uganda.

**Methods:**

Eight in-depth interviews (IDIs) were held with peer supervisors while five focus group discussions (FGDs) were conducted among registered drug sellers at the end of the peer supervision intervention. The study assessed experiences and challenges of peer supervisors and drug sellers regarding peer supervision. Transcripts were imported into Atlas.ti 7 qualitative data management software where they were analysed using thematic content analysis.

**Results:**

Initially, peer supervisors were disliked and regarded by drug sellers as another extension of drug inspectors. However, with time a good relationship was established between drug sellers and peer supervisors leading to regular, predictable and supportive peer supervision. This increased confidence of drug sellers in using respiratory timers and rapid diagnostic tests in diagnosing pneumonia symptoms and uncomplicated malaria, respectively, among children under 5 years. There was also an improvement in completing the sick child register which was used for self-assessment by drug sellers. The drug shop association was mentioned as a place where peer supervision should be anchored since it was a one-stop centre for sharing experiences and continuous professional development. Drug sellers proposed including community health workers in monthly drug shop association meetings so that they may also gain from the associated benefits. Untimely completion of the sick child registers by drug sellers and inadequate financial resources were the main peer supervision challenges mentioned.

**Conclusion:**

Drug sellers benefitted from peer supervision by developing a good relationship with peer supervisors. This relationship guaranteed reliable and predictable supervision ultimately leading to improved treatment practices. There is need to explore the minimum resources needed for peer supervision of drug sellers to further inform practice and policy.

## Background

Supervision of private drug sellers is important in assuring quality of treatment since they play an important service delivery role in low income countries, such as Uganda [1, 2]. This role encompasses the treatment of children less than 5 years of age, with uncomplicated malaria, pneumonia symptoms and non-bloody diarrhoea through strategies such as the integrated community case management (iCCM) of childhood illnesses [3, 4]. However, regulatory supervision among private drug sellers is weak since self-supervision is prescribed by policy guidelines necessitating significant investment [5, 6]. The investment would go a long way in curtailing childhood morbidities and mortalities due to malaria, pneumonia and diarrhoea which are still rampant in low and middle income countries [7–9]. Such investments would include but not be limited to use of peer supervision.

Peer supervision is a type of supervision where people of similar hierarchical status or who perceive themselves as equal encourage and enhance learning and development [10]. Peer supervision has been shown to enhance community health worker motivation, performance and quality of care when combined with supportive supervision [11, 12]. However, much of this evidence has been generated from public health settings with less data coming from private and informal health providers who play a significant role in health provision in low and middle income countries [13]. Research shows that in some contexts, about 59% of children in rural settings seek the first form of care from private health providers comprised mainly of drug shops [14, 15]. In these rural settings, access to timely and quality public health care remains a challenge [16]. As such, most deaths due to malaria, pneumonia and diarrhoea in sub-Saharan Africa, where children make up 70–90% of the population, occur in rural settings [17, 18].

In Uganda, drug shops are licensed according to the national professional guidelines for licensing and renewal for Class C drug shops by the National Drug Authority (NDA) [19]. Licensing of drug shops is done after potential drug sellers fulfill conditions related to location of the drug shop and qualifications of the person intending to operate the drug shop. Qualified personnel to operate drug shops include: enrolled nurses, comprehensive nurses, nurses and pharmacy technicians. The qualified personnel are expected to engage in self-supervision after they have been licensed. Self-supervision is largely informal, unsystematic and unprofessional because it is difficult to tell at which stage the supervisee may need help. Moreover, the hallmark of self-supervision is a lack of consulting any person which impedes learning, the heart of supervision [20]. This exposes a dynamic sector such as that of drug sellers, whose main aim is to maximize profit, to errant behaviour. On the other hand, peer supervision is premised on consulting and helps new-comers settle within a new environment ultimately executing their duties in an appropriate way in the shortest time possible [21, 22].

Through repeated networking with fellow supervisees, peer supervision is seen as one of the best ways of accessing the wisdom of others in a cooperative and mutual way providing an opportunity to refine professional skills through immediate feedback [23]. Therefore, to strengthen supervision among drug sellers, it was envisaged that since peer supervision had been effective in other settings such as mental health care delivery [24], there was a likelihood that similar results would be achieved among drug sellers in rural settings in Uganda. The piloting of peer supervision among rural private drug sellers in Luuka District was premised on the fact that that despite being licensed, receiving iCCM training and being inspected by District Drug Inspectors (DDIs), inappropriate treatment of children under 5 years continues unabated [25]. In addition, there was hardly any existing literature on peer supervision among drug sellers in Uganda. The study, therefore, assessed experiences of peer supervision among drug sellers and peer supervisors in the private sector at the end of a peer supervision intervention in Luuka District in East Central Uganda.

## Methods

### Study area and setting

**Fig. 1:**
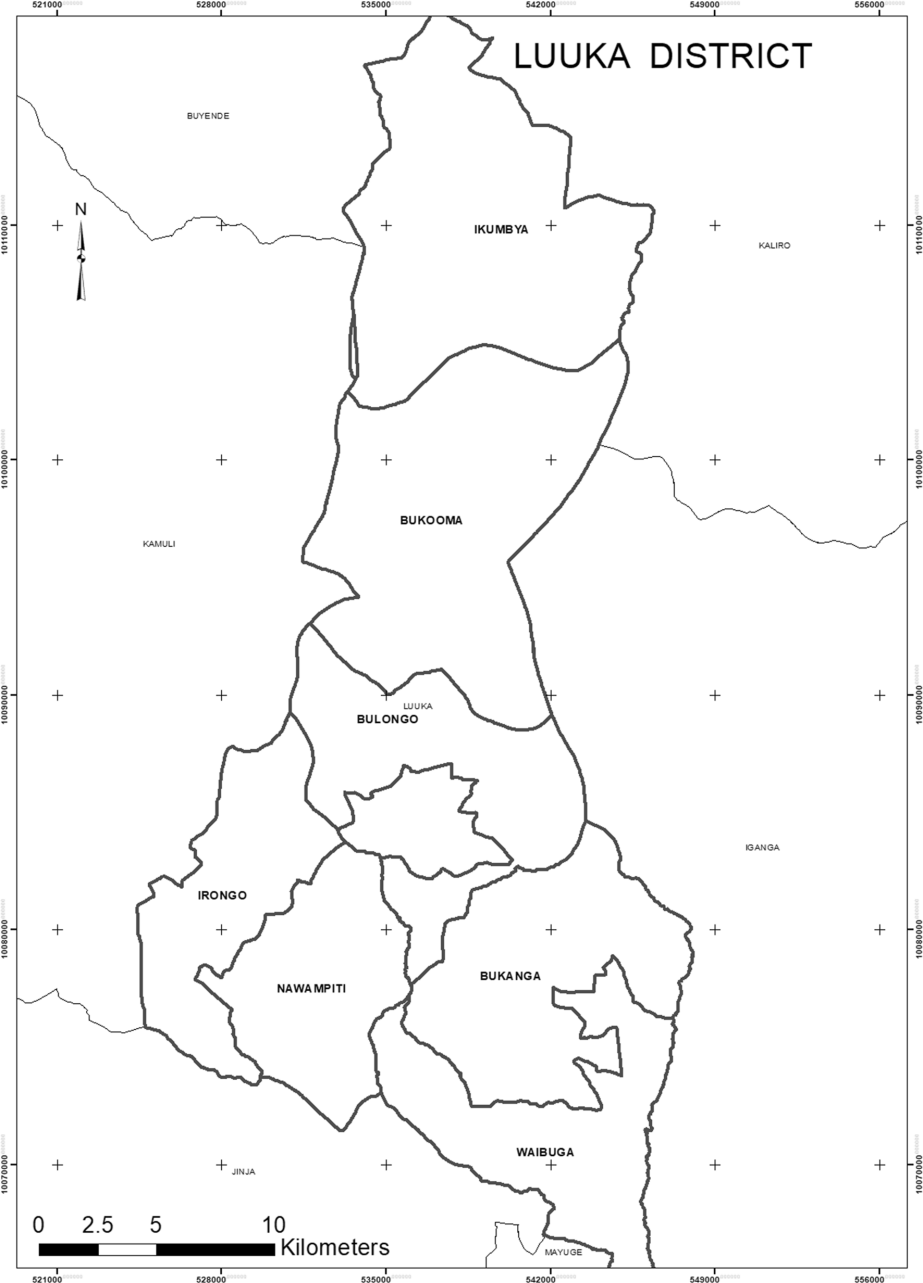
Map of Luuka District showing the study sub-counties

The study was conducted in Luuka District which is one of the 10 districts making up the Busoga sub-region in East Central Uganda. The east central region where Luuka District is located has an under five mortality ranging between 73 and 90 per 1000 live births [26]. The district is made up of 7 sub-counties (Ikumbya, Bukooma, Bulongo, Irongo, Nawampiti, Waibuga and Bukanga) and 1 town council (Luuka town council) as shown in Fig. 1.

Presently, the district has no hospital, has 1 Health Centre (HC) IV, 6 HC IIIs and 16 HC IIs. Only 61 out of the 340 villages (18%) have community health workers (CHWs) locally known as village health teams (VHTs). Up to 49% of the total population do not live within the recommended 5 km of a health facility [26]. However, all villages have drug shops where preliminary self-treatment can be sought before a health worker is seen. In Luuka District, drug shops are under a drug shop association which has a committee headed by a chairman. Drug sellers meet every month under the auspices of the drug shop association to deliberate on matters concerning licences and working conditions affecting their daily operations.

### The intervention

Lived experiences on peer supervision were captured from peer supervisors and drug sellers after the peer supervision model was tested for effectiveness on appropriate treatment among drug sellers in Luuka District. In each of the eight sub-counties that make up Luuka District, only registered drug sellers were invited for a meeting where selection of peer supervisors took place. In order for one to be considered eligible as a peer supervisor, the person must have had formal iCCM training and should have been operating a drug shop for at least 5 years. At the meetings in the different sub-counties, those who met the mentioned criteria were asked to raise their hands. Names of those whose names were raised were written on a flip chart and drug sellers were asked to second those who willingly agreed to be potential peer supervisors. There after, voting by show of hands ensued and the person with the highest number of voters was considered the peer supervisor for that sub-county. Unregistered drug sellers were not invited because they operate illegally as far as the law is concerned. In total, eight peer supervisors were selected and instructed to report to the District Drug Inspector (DDI) who derives the inspection mandate from the District Health Officer (DHO). The DDI continued the traditional inspection role as per statutory mandate during the course of the intervention.

The peer supervisors underwent a 3-day iCCM refresher training where emphasis on adhering to standard treatment guidelines was stressed [27]. Other topics handled during the training sessions included: adhering to ethical standards of supervision; reporting drug sellers who do not adhere to treatment guidelines to the DDI or DHO; and mediating disputes such as those that may arise between drug sellers, peer supervisors and district or central government inspectors. Peer supervisors were given an allowance of 80,000 Uganda shillings equivalent to 22 United States Dollars ($ 22) at the end of each month for a period of 1 year to cater for lunch, transport and other incidentals during the intervention period. The assumption was that each peer supervisor would visit all drug sellers within their designated sub-county once every month, and that supervision visits would not exceed 1 day. A total of 60 drug shops were visited by the eight peer supervisors during the study period. On average, each peer supervisor visited eight drug shops per month.

Peer supervisors were equipped with supervision checklists whose purpose was to make monthly summaries of appropriate treatment of children under 5 years from drug shop sick child registers. Peer supervisors also checked whether sick child registers were being filled by drug sellers. This way, they were able to assess whether drug sellers were adhering to the standard treatment guidelines. The peer supervisors were instructed to adhere to the highest form of privacy, professionalism, integrity, continuous learning and empathy. In the peer supervision model, the investigators worked with an active district drug shop association where many drug sellers met every month particularly to attend continuous medical education-related seminars. The peer supervision model strengthened self-supervision that is currently prescribed by policy guidelines.

### Study design, participants and sampling procedures

A qualitative study based on in-depth interviews (IDIs) and focus group discussions (FGDs) was conducted. Participants were selected based on: (1) being a statutorily licensed drug seller; and (2) being a democratically elected peer supervisor involved in the supervision process. IDIs were conducted with peer supervisors while FGDs were conducted with drug sellers. Each FGD was composed of nine nursing assistants and either a nurse or midwife. This is because the number of nurses and midwives was too small to make a group of 8 to 12 people which is desirable for a FGD [28]. As such, the five members (nurses and midwives) were placed in each of the five focus groups with nursing assistants which was aimed at creating homogeneity across groups. The IDIs and FGDs were conducted from the sub-county headquarters in the seven sub-counties. Interviews held within the town council were conducted from the town council main hall. All places were devoid of noise. Registered drug sellers and peer supervisors who were involved in the peer supervision exercise were purposively sampled and mobilized through the DDI. The DDI got official communication concerning the interviews from the DHO who was informed in writing by the lead researcher (AB).

### Data collection

An interview guide for IDIs and FGDs with the aim of capturing experiences of peer supervisors and drug sellers on the peer supervision intervention was developed by the research team. Included in the interview guides were key questions aimed at understanding how drug sellers felt about being supervised by peers and how peers felt supervising colleagues. The interview guide for IDIs explored how peer supervisors felt supervising fellow drug sellers, what they wanted improved in peer supervision, how best peer supervision and the drug shop association could be merged and which community members can be added to and benefit from peer supervision. The interview guide for FGDs assessed how drug sellers interacted with peer supervisors, to what extent the drug shop association amalgamated with peer supervision and what drug sellers wanted added to peer supervision to improve its smooth running. During indepth interviews, at individual level, peer supervisors were probed until the team lead (AB) felt that additional data collected was redundant of data already collected [29]. Focus group discussions were conducted with drug sellers until additional interviews yielded no new information a phenomenon refered to as saturation [30]. Saturation occurred with the sixth IDI and fourth FGD. However, two additional IDIs and one additional FGD were done to ensure that all information was captured and no new information was left out. Whereas the interview guide was in English, during interviews, moderators experienced in conducting IDIs and FGDs were conversant with English and Lusoga, the most widely spoken language in Luuka District. Interview questions were asked and recorded in Lusoga. Total time taken for each IDI ranged between 45 and 55 min, while time taken for each FGD ranged between 50 and 70 min. A note taker assisted the moderator in taking notes and digital audio recording conversations. All peer supervisors and registered drug sellers present at the time interviews were held participated.

### Data management and analysis

All audio recorded interviews and group discussions were translated from Lusoga to English as they were transcribed verbatim into Microsoft word documents. All transcripts were verified to be a true reflection of what transpired in the IDIs and FGDs before analysis. The translated scripts were then uploaded into Atlas.ti 7 qualitative data management software for analysis. After four IDIs and two FGDs were independently coded by two researchers (AB and MM), a discussion with intention to agree on how to resolve differences as well as improve validity and reliability of developed codes ensued until consensus was reached. The agreed upon codes were then used to code the rest of the transcripts. A similar procedure of agreeing upon sub-categories, categories and themes was followed to address issues of rigour. In both instances, the research team ensured that it achieved inductive thematic saturation, that is to say, new codes and themes were redundant of codes and themes already constructed. Graneheim and Lundman’s framework for capturing both latent and manifest content in transcripts was used for this content thematic analysis approach [31]. During analysis, sub-categories were derived from codes. Codes were derived from unifying meaning units abstracted from condensed meaning units. Abstract level categories were then formed out of sub-categories with similar meaning [31]. In the final analysis, a larger narrative of themes from condensed categories were developed. To address rigour and trustworthiness of the qualitative data collected, a dissemination meeting was held with participants after the interviews were done to confirm whether it was reflective of what was discussed.

## Results

A total of 59 people participated in the study. Of these, 51 were drug sellers (10 male and 41 female) while the remaining 8 were peer supervisors (7 male and 1 female). The mean (SD) age of the drug sellers was 31.2 (7.7) years while the mean (SD) age of the peer supervisors was 38.5 (9) years. A majority of the drug sellers (n = 46, 90.2%) and peer supervisors (n = 5, 9.8%) were nursing assistants. The study endeavoured to understand how drug sellers and peer supervisors experienced the peer supervision process and challenges encountered. Four major themes emanated from the study. These were; Favourability of supervision, pereceived benefits from supervision, supervision structure and challenges of supervision.

### Favourability of supervision

Traditionally, drug sellers were used to self-supervision following a successful licensing procedure. With the piloting of peer supervision, drug sellers in majority of the FGDs said that peer supervisors were good to them because they made regular, predictable support supervision visits to their drug shops. The drug sellers also mentioned that since the peer supervisors were chosen from amongst them, they had developed a good working relationship with the peer supervisors who were more of acquaintances familiar with the behaviour and context in which drug sellers operated. This good relationship diminished the fear that was associated with government inspection.

*“Way back, we never used to be so free with them[drug inspectors] but now, we don’t fear them. You can explain to them anything. But the other time, there was a lot of fear.”* (Participant 1, FGD 1, Nursing assistants)

*“Me what I saw in these peer supervisors of ours was the issue of not taking long to come. I know that at times they also don’t have the means but they endeavour to check on us more regularly when they can.”* (Participant 3, FGD 3, Nursing assistants)

Peer supervisors revealed that initially, drug sellers thought that peer supervisors were another extension of drug inspectors because each sub-county had a peer supervisor. As such, whenever there was a round of supervision visits, some drug sellers opted to shut their drug shops because traditionally, many inspection visits are characterized by government inspectors confiscating drugs from drug sellers which disorganizes service delivery. In addition, peer supervisors said that drug sellers had a perception that they were being paid a lot of money and yet as far as they were concerned, they were making a lot of sacrifices for the better running of drug shops.

*“At first, they [drug sellers] thought that maybe we [peer supervisors] were actually sent from the drug inspectors office since wewere trained in a big group. Some people did not really take note of our faces and therefore could not identify us as their selected supervisors. So, they would never meet us and when you would go to meet them, they would just run away.”* (In-depth interview, Female, Enrolled Nurse)

In addition, most peer supervisors said that much as they cross-checked drug shop sick child registers, they were cognisant of the fact that they were unable to punish errant drug sellers. As such, they mentioned that peer supervision would be adequate if it were supplemented with inspection. Drug inspectors have the ability to apprehend drug sellers in possession of injectables and other drugs not allowed by policy guidelines. In addition, peer supervisors said that they noticed the community prefered drug sellers that had been peer supervised and inspected. This is because the community was aware that government inspectors were always on the look out for drug sellers that were duly qualified and adhered to policy guidelines. Therefore, drug sellers that were peer supervised and kept their drug shops open during inspection visits were highly respected by community members.

*“They [drug sellers] will do shoddy work when nobody inspects them and will be in possession of drugs they are not supposed to have. But if the drug inspector regularly visits them, it can strengthen the law of not having drugs that they are not supposed to have …….. When the community sees inspectors visiting drug sellers, they always go where they have inspected because they know they have the right drugs.”* (In-depth interview, Female, Nursing Assistant)

### Perceived benefits from supervision

Majority of the drug sellers in the FGDs said that peer supervision had increased their confidence in treating children by being reminded what they may have forgotten. In addition, drug sellers said that peer supervision not only helped them to improve treatment but also validate them as health workers because peer supervisors endeavoured to visit when drug sellers had some patients. The cordial questions asked by peer supervisors regarding use of the thermometer, completing the sick child register, and ability to use both the respiratory timer and malaria rapid diagnostic tests gave patients a good impression of the drug sellers. As such, drug sellers reported that peer supervision had helped their businesses grow. Drug sellers also highlighted the importance of the drug shop sick child registers. They said that the registers helped them in self assessment by knowing how many and how well children were treated the previous month. The assessment was used as a benchmark for appropriate treatment of children in the subsequent month.

*“We no longer fear[treating children] because we know that what we do is right. Nowadays, we treat with the full dosage and they [patients] get well and your business moves on, and even the patients are happy that they are healed and they will continue to come back*.” (Participant 1, FGD1, Nursing assistants)

*“The good things we have got from them [peer supervisors] …….those registers [sick child registers] they gave us have helped us because you can go through them and know that last month I treated this number of children, I took like this number of blood samples, how many had malaria and you know that.”* (Participant 2, FGD3, Nursing assistants)

In-depth interviews with peer supervisors revealed that most of them were involved in reminding drug sellers about the value of appropriate treatment of children. This, they said drawing from their past experience where they did not know how to treat children under 5 years until they got some form of medical training. With some additional training in peer supervision, peer supervisors felt even more knowledgeable in managing febrile illnesses including referring complicated cases to higher level health facilities. This knowledge was shared with drug sellers during supervision visits. The acquired confidence from the peer supervision training also helped the peer supervisors to liase with and be recognized by the higher level health facilities. Peer supervisors also said that the drug shop sick child registers were a good innovation because they helped keep record of the number of children that had been treated by drug sellers. In the past, there was no record of children treated and, therefore, it was very hard to understand who was offering the right treatment and who was not. As such, the registers made supervision and the targeted counselling easy and feasible.

*“Back then, a sick patient would be brought in and you do not know what to do but now I know how to deal with them. We even have chances of referral. At the moment we are well known by the big facilities which was not the case before.”* (In-depth interview, Male, Enrolled Nurse)

*“The good thing is that ever since the exercise began, many [drug sellers] didn’t have drug shop sick child registers. Ever since then, their cases whether pneumonia or diarrhea or malaria, are registered. So, that’s the benefit.”* (In-depth interview, Male, Enrolled Nurse)

### Supervision structure

Drug sellers were also asked to share experiences on how they felt the drug shop association had combined with peer supervisors, and whether this amalgamation was helpful. The majority of the drug sellers in the FGDs said that initially, the meetings held every end of month with drug sellers, peer supervisors and members of the drug shop association were useful. This was because during these meetings, drug sellers and peer supervisors shared experiences which helped to empower everyone. During FGDs, peer supervisors were asked about the personnel they felt should have been part of the peer supervision process but missed out. Majority of the participants across all the five FGDs groups mentioned VHTs because all villages in the district have them. The drug sellers felt that if they combined with VHTs, this cadre would be very instrumental in organizing meetings for themselves at village level ultimately benefitting from the peer supervision process.

*“Every time we meet as the association, we also meet and sit down with the peer supervisors and share knowledge with each other about how the work is going on in their various locations of supervision. So, if one is having difficulty in a sub county, they are empowered to continue with the work.”* (Participant 6, FGD4, Nursing assistants)

*“Am supplementing on sensitizing the community. We can unite with the VHTs who can mobilize for meetings in their own villages. At these meetings, we can all benefit from health education which can be facilitated by the peer supervisors who can come into join the community and health workers.”* (Participant 8, FGD2, Nursing assistants)

Information gathered during IDIs with most peer supervisors showed that drug sellers were very receptive to peer supervisors during visits. Peer supervisors felt that drug sellers were particularly welcoming because the peer supervisors were structurally at a higher level even though they were colleagues. In addition, from the peer supervisors’ perspective, drug sellers were happy to be visited by peer supervisors because they felt the peer supervisors were more knowledgeable. To this effect, even though there was knowledge being exchanged, the drug sellers benefitted more. Peer supervisors were also asked who else should be involved in the peer supervision process. Majority of the peer supervisors responded by saying that they felt VHTs were a missing component of peer supervision. The peer supervisors said that since VHTs were already part of the communities being supervised, extending supervisory services to them would play a very big role in making sure more people benefitted from peer supervision.

*“Now in most cases when we are supervising, those we supervise do respect us. Also they feel good especially if the supervisor coming is more high ranking than them. They feel happy because they expect to learn more than what they already learnt”.* (In-depth interview, Female,Nursing assistant)

“*As a community for instance, where I live, we use VHTs. When they come, we teach them for example about diarrhea: what causes it and how it is spread, then we also teach the mothers in the community how to prepare ORS, and teach them how to administer the right dosage of zinc for a child. We also teach them how to clean and maintain the hygiene of the environment the children live in.”* (In-depth interview, Male, Enrolled Nurse)

### Supervision challenges

Peer supervisors cited a challenge of not filling in the sick child register in a timely manner. This challenge was brought about by many drug sellers not being conversant with spelling clan and or family names of the children that came to the drug sellers for treatment. Some drug sellers were said to fill in sick child registers a day before they knew that the peer supervisor would make a supervision visit. In other instances, drug sellers even filled in the sick child registers when the peer supervisors were at the drug shops already. When pressed to explain why the habit of not completing the sick child registers had persisted, some drug sellers were quick to say that they did not get any children seeking treatment on those days. However, while some rows in the sick child register had treatment data, the names and ages of the children were missing. This slowed the supervision process.

*“The drug sellers don’t write anything [in the sick child register] and the day they know you will be supervising is when they write. When you ask about the previous days, they tell you they didn’t get any patient in between and at times when you passby you see them treating. The problem is that some drug sellers don’t know how to write the clan names[sir names of children]. So, they only write the first names.”* (In-depth interview, Male, Enrolled Comprehensive Nurse)

Both drug sellers and peer supervisors said that the peer supervision process would have been a lot more successful if peer supervisors had adequate transport to enable the peer supervisors visit every drug shop within their jurisdiction. This is because most peer supervisors were using bicycles. The bad terrain and harsh conditions during the rainy season made bicycle manouvers slow ultimately leading to supervision delays. In some instances, peer supervisors had to hire motor bikes using personal resources to ensure that supervision was done. This greatly impacted on their monthly allowance.

*“We get difficulties in transport because the drug shops are not here. There is some kind of distance and some of us fail to catch up with time because of the hardship of transport. You may want to reach at 10am and you find yourself reaching at 11am and where it is very far you may fail to go as many times as you want.”* (In-depth interview, Male, Enrolled Comprehensive Nurse)

## Discussion

This study piloted peer supervision in response to the absence of human resources for health involved in supervision of private drug sellers in rural Uganda. Overall, four themes emanated from the study. These are: supervision practices, treatment practices, supervision structure and challenges experienced during peer supervision. The findings of the study show that peer supervision was predictable, improved the confidence of drug sellers in treating children and may be viable if anchored in a recognized supervisory structure. These findings are expected to not only inform future practice and policy but also other interventions and studies aimed at exploring peer supervision among drug sellers in Uganda and other low and middle countries.

Results from this study revealed that after piloting peer supervision, both drug sellers and peer supervisors felt that peer supervision was reliable, regular and predictable. These results are similar to those from India where peer supervisors were able to reliably assess the quality of a therapy just as experts did [24]. However, while an amalgamation of peer supervisors with experts was preferred in India, in Luuka District, peer supervisors preferred an amalgamation with drug inspectors for quality assurance purposes. This implies that whereas peer supervision may involve lay providers who can be trained, there is need to have experienced personnel at the helm of the supervision structure to provide the much needed guidance and feedback where necessary.

Study results also showed that both peer supervisors and drug sellers talked highly about how peer supervision had improved confidence in diagnosis and treatment skills of drug sellers. Studies in low-income countries aimed at delivering mental health care have reached similar conclusions [32, 33]. In these studies, peers were able to detect, diagnose and treat mental disorders thus reducing the burden of taking care of patients from more experienced health workers and care givers. In the pilot study carried out in Luuka District, peer supervision instilled confidence among drug sellers enabling them to offer appropriate treatment to children under 5 years of age similar to what they would get from public health facilities. This is commendable because public health care in Uganda experiences numerous challenges such as inadequate human resources for health which would endanger the lives of patients if private health providers were absent [34, 35]. The confidence instilled in the drug sellers had a lot to do with the caliber of peer supervisors chosen. During selection of peer supervisors, the investigators of the study were aware that beyond academic qualifications, a good supervisor ought to have a strong desire to be appreciated and recognized as a good supervisor, have strong self-motivation as well as high self-confidence [36]. However, because these attributes were not easy to tell from the people chosen, the investigators relied on the processes used during the selection of peer supervisors to go passed this impasse. For instance, all drug sellers that allowed secondment by fellow drug sellers were presumed to have high confidence and a strong desire to be appreciated and recognized which are important supervisor attributes. Relatedly, the importance of the drug shop sick child register in improving treatment was emphasized by both drug sellers and peer supervisors. In this pilot study, peer supervisors monitored the kind of treatment given to children under 5 years of age. This was used as a form of assessment and quality assurance process. Elsewhere, records have been used in health care settings for assessment and quality assurance just like this study [37].

Evidence from this pilot study showed that both drug sellers and peer supervisors used monthly drug shop association meetings to share experiences. These meetings were also used as a form of continuous professional development. Hence, anchoring the peer supervision intervention in a district structure uniting all cadres involved in the sale of drugs can be beneficial if experiences are shared. This finding collaborates with evidence from systematic reviews on training of health workers in evidence based practice [38]. The systematic reviews note that for health workers to deliver services reliably, programs must incorporate training, supervision and mentorship. The aim of incorporating these three attributes in any program is to ensure health workers remain abreast with topical solutions to prevailing challenges. Such is the intersection shared with the drug shop association in our study.

In addition, drug sellers said it would be beneficial if Community Health Workers (also referred to as VHTs), who are the first level of government health care, were part of drug shop trainings. This is because both cadres receive parallel training yet treat the same ailments as part of their responsibilities. Moreover, VHTs do not have an association at district level which puts them at a great disadvantage. Thus, if the trainings included VHTs, this would improve health care in the district. Studies have showed that initial trainings given to health workers are good but will not build confidence and competence if not sustained [32]. Findings from this study reveal that the drug shop association is a structure that can be explored to ensure sustainability. This is because the drug shop association is managed by elected members from amongst the drug sellers who strive to ensure that the association thrives and helps members to combat any arising challenge. Therefore, the drug shop association is worth exploring for delivery of more sustainable interventions including supporting community health workers in their role of iCCM at village level.

A major concern from the study on experiences of peer supervision was complacency of drug sellers resulting in untimely data completion. Peer supervisors noted that because drug sellers had become used to the supervision process, some were not keen enough to complete drug shop sick child registers immediately after treating children with febrile illnesses. More so, other drug sellers preferred to complete registers on the day peer supervisors made visits to drug shops which many times wasted time of the peer supervisors. Complacency may have arisen as a result of peer supervisors seeking to have a good relationship with drug sellers consequently leading to underperformance. Findings from this study are similar to what human relation experts have noted with supervisors who focus on nurturing bottom-line relationships [39]. Quite often, such supervisors are perceived as low quality leaders by supervisees hence supervisees may respond by underperforming. It is, therefore, advisable that supervisors have a standard mode of operation stipulating expectations of the supervisor and supervisee as this will minimize nurturing of bottom-line relationships.

Peer supervisors also felt that there was need for improvement in the resources they were given for supervision. They said this because most of the peer supervisors used bicycles to manoeuver difficult terrain which was worse during rainy seasons. Those without bicycles used their monthly allowance to hire motor bikes leaving them with little money to spare. This was a pilot study aimed at understanding whether peer supervision was feasible among rural private drug sellers, and if so, how it can be conducted. It was conducted on a small number of drug sellers with intention to inform quality and efficiency during scale up. Pilot studies are faced with many challenges including estimating with certainty the amount of resources that may be adequate to ensure smooth execution of interventions [40, 41]. Similarly, it was hard to estimate the amount of financial resources to avail peer supervisors for smooth running of peer supervision in this pilot study.

The major strength of our study is that there was two-way sharing of knowledge between drug sellers and peer supervisors. In addition, there were improved treatment practices by drug sellers without any expert supervisors other than fellow drug sellers. Whereas peer supervisors lamented about financial resources availed during the piloting of peer supervision, the design and resources provided were justified. Future studies among drug sellers being supervised by peers with government support are required. However, such studies may only be possible when peer supervision is adapted as a formal method of supervision for private drug sellers.

## Conclusion

Drug sellers benefitted from peer supervision by developing a good relationship with peer supervisors. This relationship guaranteed reliable and predictable supervision which contributed to improved confidence of drug sellers in offering appropriate treatment to children less than 5 years of age. More studies on peer supervision are needed to further explore the minimum resources needed for adequate peer supervision of private drug sellers. There is also need for further research on how completing drug shop sick child registers by drug sellers can be improved.

## Data Availability

Datasets used during the study are available from the corresponding author on reasonable request.
